# New York Odontological Society

**Published:** 1891-01

**Authors:** 

**Affiliations:** New York Odontological Society


### NEW YORK ODONTOLOGICAL SOCIETY.

THE New York Odontological Society held its regular monthly meeting, Tuesday evening, November 18, 1890, in the New York Academy of Medicine, 17 West Forty-third Street.

The president, Dr. J. Morgan Howe, in the chair.

### INCIDENTS OF OFFICE PRACTICE AND CASUAL COMMUNICATIONS.

*Dr. Allan.*—Mr. President, I have been experimenting for the last month or so upon the vexed question of filling the canals of pulpless teeth with an entirely new preparation and by a new method. Of course, I cannot say at this date whether I am going to obtain any great amount of success or not, but the results promise so well, and the materials that I use seem to be so well adapted to the purpose, that I thought, even at this early stage in my use of them, I would bring it before the society for criticism, or for any remarks that may be made upon it. The points to be aimed at in filling a canal are, first, that the material used shall be negative in all its reactions; second, that it shall be easily and readily adapted to the walls of the canal. A large number of materials have been used that have had their day and passed away. The number of discussions in the journals, if we will read them, show how large a latitude there is for materials, and how many and various are the ways employed, and we must acknowledge that *the* method of filling root-canals is yet to be found out; and I look upon this method that I have adopted as only a step that I hope will be in advance. It is this: I take nitro-cellulose, which is negative in all its reactions, which is not a material that would be acted on by any of the fluids of the mouth, or any fluid that would be found in the pulp-canals, and I dissolve it in two parts of ether to one of alcohol. It is admissible in all proportions. I dissolve enough nitro-cellulose in the mixture to form a thick paste, and having obtained this mixture and gotten my tooth ready for filling, the rubber dam applied, and the root thoroughly dried out and cleansed, I first wipe it out with a mixture of two parts of ether to one of alcohol. I then take a small pledget of surgical cotton that has been soaked in a solution of one part to a thousand of bichloride of mercury, and dip it in that solution, carefully wiping off any surplus, and this cotton, wrapped around a fine broach, can be passed up into the finest canal. It readily adapts itself to the walls of the canal, and, so far as I know, makes a perfect root-filling. On top of this I place ordinary gutta-percha, which, I think, takes up any little excess of the solution that the nitro-cellulose is dissolved in. I do not see any objections to this method of practice. If there are any, I would like to have them pointed out. It certainly seems to be satisfactory, and places in the root-canal an absolutely negative material, one that germs of any kind cannot act upon, and which adapts itself perfectly to the walls of the canal, and fills all the requirements of such a filling better than anything I have heretofore adopted. I will be glad to hear any criticism that members may have to make upon it.

*Dr. Charles W. Miller.*—Does Dr. Allan fill the entire canal with nitro-cellulose?

*Dr. Allan.*—Yes, the entire root-canal and the pulp-chamber. I have taken pieces of antiseptic cotton and adapted them to the walls of a canal in this method, and then withdrawn them and allowed them to dry, and I find that the shrinkage, if any, is comparatively slight, and the resultant is in an hour or so hardened, and apparently has taken the shape and contour of the canal perfectly. Nitro-cellulose is the base from which celluloid is made.

*The President.*—Gentlemen, we will listen to the report of the committee on the death of Dr. Bronson.

The secretary read the following report:

WHEREAS, The sudden and unexpected death of our late associate and fellow-member, DR. WILLIAM A. BRONSON, has bereft this society of one of its most respected and best esteemed members,—one who labored assiduously for the welfare of the society since the early days of its formation, and who has filled with credit and ability every position of honor that the society could bestow upon him; therefore be it

*Resolved*, That we desire to express our appreciation of his valuable services to this organization as an earnest co-worker, and of his delightful companionship as a fellow-member.

*Resolved*, That in his exemplary life we recognized a true and noble character; with a disposition kind, considerate, and devoid of selfishness; conscientious to an exalted degree; just and generous in his dealings with others; modest and gentle in all his ways.

*As a professional man* he occupied a prominent position in his specialty, possessing sound judgment, combined with excellent manipulative ability, admirably fitting him for his calling.

*As a member of this Society* he was almost invariably present at its meetings, and in them he always manifested an exceeding interest. To each and every member he was a warm and sincere friend, ready to aid and advance their interests to the best of his ability.

*Resolved*, That while we mourn and deplore the departure of this sincere friend and beloved member, we reverently thank God that we so long enjoyed his companionship. And in breathing our farewell benediction, these fitting words of peace and promise come to our minds: "Well done good and faithful servant, enter into the joy of thy Lord."

CHAS. E. FRANCIS.
CHARLES MILLER.
S. G. PERRY.

On motion of Dr. Abbott, the report was adopted and ordered to be spread upon the minutes of the Society, a memorial page to be set apart for that purpose.

*The President.*—Gentlemen, I have now the pleasure of introducing to you Professor D. Bryson Delavan, of the New York Polyclinic, who will read a paper entitled "The Influence of Adenoid Hypertrophy at the Vault of the Pharynx upon the Development of the Hard Palate."

(For Dr. Delavan's paper, see page 1.)

*Dr. Delavan.*—I have asked several patients to come here this evening in order to exhibit these different things to you, but, with two exceptions, they have not appeared. I have a young gentleman patient here in whose case is shown the very point that I tried to emphasize in my paper,—namely, that while the work of the dentist may be remarkably perfect in its way, as in this patient, the deformity of the hard palate persists in spite of the good work done by the dentist. In this case (referring to patient present, a boy of seventeen years) the teeth have been very well regulated. I do not think I ever saw a finer result; but you see that the arch is very high still, and that has remained in spite of the improvement in the teeth. If any gentlemen care to look at this case they will see a fine illustration of the main point of the paper. My young friend says he had a cast of his teeth as they were. I understand the deformity was very great. Who was your dentist?

*The Patient.*—The dentist who regulated my teeth is Dr. S. H. Guilford, of Philadelphia. I could get my thumb in between the teeth of the upper and lower jaws. Now I can get my lower teeth out over the upper ones.

*Dr. Delavan.*—This young woman (referring to another patient) suffered severe symptoms from nasal obstruction due to adenoid hypertrophy; she had also enlarged tonsils. The high arched roof is here well represented.

*Dr. Jarvie.*—Are both these cases of adenoid hypertrophy?

*Dr. Delavan.*—They are.

*The President.*—Gentlemen, the subject of this very interesting paper by Professor Delavan is before you for discussion. I wish to express, what I know is in the minds of all, that we are under great obligations to him for bringing the subject before us in this graphic and clear manner, and suggesting to us causes of a difficulty, the etiology of which has been to us obscure. No longer ago than last January Dr. E. S. Talbot, of Chicago, read a paper in this city, in which, I believe, he opposed the idea that mouth-breathing could produce any effect upon the shape of the hard palate. The subject was considerably discussed by those present; Professor Peirce, of Philadelphia, alone, I think, of those who discussed it, taking the view that mouth-breathing could alter the form of the hard palate and the upper arch. It seems as though this suggestion and explanation of the causes that may operate to produce a high vaulted arch with narrowness opens up for dentists a new field of thought and investigation. I hope that you will discuss the paper as promptly and concisely as possible. Dr. S. H. Guilford said, at the time of the discussion of Dr. Talbot's paper, that he believed there was "less known of the causes producing the V-shaped maxilla than any other form of irregularity with which we have to deal."

*Dr. E. S. Niles.*—I feel very thankful to Dr. Delavan for the presentation of this subject. It was called to my attention some two years ago by a mutual friend of Dr. Delavan and myself in Boston,—Dr. Hooper. At that time two cases came to me for treatment of irregularities, and they were also under treatment for adenoid vegetations. The irregularity of the teeth was quite marked, and while I succeeded in correcting their positions, the deformity of the hard palate remained as it was. I believe this subject is a key to the solution of the question of the cause of a great deal of the deformity and irregularity which we meet with in practice hitherto unexplained. This deposit or growth taking place gradually from very early childhood, at a time when the hard tissues are quite soft and but partially formed, an arrested development naturally occurs on certain lines of normal growth. This would doubtless be produced mechanically in some degree by the attempt of nature to bring the teeth into position. We know that at a very early age not only the deciduous teeth but also the crowns of the permanent teeth are crowded in one over the other, and in turn seek to find their place in regular order. Nature's forces bring them gradually into position at appointed times. The difficulty in breathing owing to the arrested development of the hard tissues forming the walls of the air-chambers, we can see, would retard nature in certain directions, and bring the teeth into an irregular position, deforming the tissues surrounding them. We see, gentlemen, the subject is a new and important one for us to consider. I have some fifteen models, taken during the past few years, of cases which prove all that Dr. Delavan has so well explained to us; and, as I think over the matter, I find there is a new light in which irregularities are to be considered. In all the cases of this kind which appear in my practice I have felt that it was my first duty to refer them to specialists in this line for examination and treatment, if necessary, so that my work may be more permanent and more easily accomplished.

*Dr. Ottolengui.*—Mr. President, the gentleman who last spoke said that he always refers these cases to specialists for treatment, and the essayist did not tell us what that treatment is. I think we would be grateful to him if he would tell us how these growths are removed, that we may possibly get to be specialists ourselves in this direction.

*Dr. W. H. Dwinelle.*—I have heretofore stated my conviction that the reflex action and mechanical effects upon the teeth and jaws from adenoid vegetation in children, thereby closing the nasal passages and compelling mouth-breathing exclusively, was a subject which, as dentists, merits our serious attention. I have also referred to the fact that the naso-pharyngeal cavity obtains but small recognition from the dentists, whereas, it is often the almost exclusive region from which the most serious deformities of the teeth and jaw have their origin.

I have advocated the theory that mouth-breathing was generally caused by adenoid obstructions, and that the continued strain upon the plastic superior maxilla was often, if not generally, responsible for irregularities in that region; the remarkable coincidence occurring between mouth-breathing and the V-shaped jaw would seem to confirm the doctrine. George Catlin, the great Indian painter, in his "Breath of Life," written many years ago, wherein he deprecates mouth-breathing, attributes the narrow V-shaped jaw, the irregular, projected, and uncovered teeth, to this cause, though he did not go back far enough to trace it.

I congratulate myself, too, that the essayist of the evening, and one from a kindred profession, has so fully confirmed all that has been said in favor of the theory of the consequences of mouth-breathing.

He has described in the most lucid manner the proper method of operating. He finds a few instruments only necessary, generally the Lowenbergh forceps and the curette, assisted by sharpened finger-nail as well as the artificial nail, a metallic shield with a rounded edge mounted on a thimble similar to instruments in use with us. Still, for complex operations, the loop, the scissors, the adenotome, the mouth-gag, and retractor find their uses.

I had the pleasure of listening to a distinguished gentleman a few days ago, who says that he oftentimes removes adenoid growths with his finger and finger-nail alone. I have removed them with scarcely better facilities. There is a wonderful coincidence between the V-shaped jaw and adenoid growths. It is shown in this case which I now present to you. This is a cast of a young lady's mouth when she was about twelve years of age, and came to me for treatment. She had a V-shaped arch. It was utterly impossible for her lips to cover her teeth, which projected as you see. Her mouth drooled and was disfigured generally. I removed the adenoid growths without any trouble. She had great difficulty in breathing, could only do so through her mouth, the nasal fossæ being closed entirely, and her voice was thickened and nasal in tone. This second model shows the condition of the arch after the removal of the adenoid growth. The teeth had been regulated, and you will perceive a great change has taken place. There is not only a change in the expression of the mouth, but the position of the upper lip is much altered, as you will see. From its being a very short and very narrow upper lip, it is now a long and broad one, and so, too, I have changed the whole character of the arch, and given a clear and resonant character to her voice. Although I resorted to the usual methods for correcting the deformity of the jaw, still I think the removal of the adenoid growth had much to do with the success of the operation.

When the removal of adenoid growths are successfully performed, the changes that take place in the condition of the patient are always of the most remarkable character; according to Dr. Hooper, the whole expression of the countenance changes. The mouth generally remains closed as soon as the air-tract is made free, and the lines which have been formed at the angles of the mouth, nose, and the corners of the eyes, by the weight of the hanging lower jaw, disappear rapidly. We notice, too, the remarkable change in the quality of the voice. If these changes are so noticeable when adenoid growths are simply removed, how much more so are they when the teeth are also regulated and brought into their circle, the narrow V-shaped arch expanded to its normal breadth, the antrum, the sounding-board of the voice, is uncovered to exercise its function, and the remodelling of the face at large has carried it back to its original type.

*Dr. Jarvie.*—Mr. President, I do not propose for a moment to discuss this paper. There are some matters connected with the subject that I do not feel competent to discuss; but I have been a most attentive listener, and have been very much interested in it. Some thoughts arise in my mind in connection with this subject of which it may be in place to speak here. First, the concurrence of the high vault and narrow arch with mouth-breathing. Whether mouth-breathing is the cause of the high vault and narrow arch I am not even yet prepared to say, but that we frequently see the two in the same person is a fact. The essayist tells us that these adenoid growths commence at a very early age, long before the bones of the hard palate have become firmly ossified, and that these growths have an influence in the shaping and thickening of the hard palate. If that is the case, by the time we as dentists see these cases it is too late to change the form of the roof of the mouth. We may change the arch, but we cannot change the roof and the high vaulted condition to any but a very limited extent, because, as the essayist tells us, the bones are thickened very much. It would therefore seem to be the place of the physician to notice these conditions of mouth-breathing, and treat the case at once, for by the time the cases get into the hands of the dentists it is too late.

While I am not prepared to oppose the theory of the essayist in regard to the mechanical causes of the high vault and narrow arch, such as the dropping of the lower jaw and the tongue lying on the floor of the mouth, I do not see how the tongue in this position can narrow the arch, though it may have an influence in causing the broadening of the lower jaw, which generally accompanies the high vaulted roof.

*Dr. Delavan.*—That is what I meant.

*Dr. Littig.*—If the theory in regard to the expansion of the lower jaw is correct, why is it not correct also that the removal of the pressure of the tongue from the teeth of the upper jaw has allowed that arch to become narrower. Is not this the primary cause,—the narrow upper arch not being due so much to the muscular action of the dragging of the lower jaw upon the cheek-muscles as to the absence of muscular action from the tongue not being in position to press upon the teeth? It has been my belief that such was the case. When I find a case where I think there is adenoid tissue I almost always send the patient to a specialist to have that tissue removed, so as not to have muscular action fighting against my efforts all the time.

*Dr. Niles.*—I would like to ask Dr. Delavan what his experience is in regard to the return of the adenoids after they have been once removed?

*Dr. Delavan.*—It very rarely—I returns, may say never,—if the hypertrophy is thoroughly removed.

*Dr. Niles.*—I think effectual treatment requires a very thorough removal of adenoids. It is customary in Boston for both medical and dental practitioners to refer patients to Dr. Hooper or some specialist in this line, who has had a great deal of experience in the removal of these growths. Last winter Dr. Hooper, I understand, operated upon some two hundred patients for this trouble alone. I think it is a very important operation, and requires great care when thoroughly performed. If there is extensive adenoid growth, the patient may have to be etherized, and we all know that operations in the vicinity of the pharynx are dangerous when the patient is thoroughly etherized, from the liability of the blood to get into the trachea, and I should hesitate to take the risk of such operations in my practice when others have better facilities for performing them. Dr. Hooper and others have devised instruments for the removal of growths and for cutting or scraping the parts that could not as well be accomplished, as Dr. Dwinelle has suggested, with the finger-nail.

*Dr. Atkinson.*—I am not happy to stand in the position I do at all, because I know the difficult task I have before me of trying to make myself understood as I mean. If I were to say with child-like simplicity what I have in mind just as it comes to me, it might seem to be very severe. The question has almost gone by default, as against the dentists; yet we all know more on this subject than any man has manifested to-night. I do not expect to be helped very largely by simple references to our reaching after cause. That seems to have been in the mind of every one who has spoken; the etiological character of the trouble seems to have been the onus bearing upon each one's mind, and of which we know next to nothing. One gentleman spoke of the bones being thickened and becoming enlarged by reason of the obstruction of nasal breathing. Do we know anything of the morphology of bones? What is the first stage of the long bones? What is the process of reducing the callous that unites a fractured long bone? It is the boring out of the medullary canal and establishing a canal inside of the bony shaft. No one seems to have thought of that at all. I want to say that I pity the man who will shed blood in dealing with adenoid growths; hence the removal of such growths with the fingers or with instruments is the making of one's self a lieutenant of his Satanic Majesty. I thought I had delineated these cases sufficiently before dentists to have them at least comprehend the first line or the letter "A" in the alphabet of the histology and embryology of the subject. This is too long a subject, and too deeply involved, to profitably speak upon without getting either heart-broken or mad.

I am not capable of being angry any more. I can have my feelings hurt, but I cannot get angry. I have had so much cause to fight the assumption of knowledge where it was not properly held in possession by those assuming it that I have learned to be very patient and very tremulous and very much agitated whenever I look over these questions. Before this Society I have delineated a case where more than thirty operations were made for adenoid growths, upon the superior maxilla and the soft palate, upon the alveolar ridge, the uvula, and behind the curtain of the soft palate, up into the upper air-passages, and down to the epiglottis, and into the upper end of the œsophagus. Thirty operations were made, simply drawing together the albuminous epithelium covering the papillæ and wetting them with a saturated solution of salicylic acid in ninety-five per cent. alcohol until it was cooked like the white of an egg. This case was one where an upper set of artificial teeth had been worn for years. It was treated once in two to four days, I think; sometimes every day. The growth was removed successfully, and the casts which I have to show you will, to any man who manifests ordinary intelligence in such matters, speak for themselves. The best diagnosis is to let the case tell its own story.

In operating in that way, be careful not to pull off the eschar of salicylate of albumen as it comes there. That which came from the soft palate and the uvula was so soft that I could not preserve it. I have some thirteen of the casts left. Each time the eschar would be removed it was dressed until it was white with the salicylic acid, then plaster of Paris was carried up and allowed to flow over until it completely reproduced the coaptation of the plate against the roof of the mouth. Each time the plaster cast was removed it was set aside, the parts dressed, and plaster again applied. I saw the red eyes of the papillæ, but I did not see a drop of blood in the whole nine or ten weeks occupied in the operations. I have repeated this so much, and with so little interest manifested, and so little comprehension, further than a little flash for the time being, that I am almost discouraged. I pity the man who uses instruments to remove adenoid growths from any portion of the air-passages. You should never make the blood come. You should never go below the epithelium, the Malpighian layer. You should never take that away. If you do, you have made it impossible to get the best results. I need only refer anatomists to the fact of the Malpighian layer being built up by, first, globular, then cylindrical, then cuboidal, flattened, squamous, pavement epithelium, until you come to the thinnest on the surface, to let them understand that all that needs to be taken away, all that can profitably be taken away, is the mucous follicle, and not to go into the connective tissue constituting the corium of the skin. The connective tissue and the blood-vessels should never be interfered with.

I have not seen a man show a mental grip of this subject to-night that you could not get by simply listening fifteen minutes before a specimen. I will be happy to show any man this case that I speak of, because I am so sure it is the biggest thing ever done on the planet respecting this kind of surgery that I am very much in earnest about it.

If you have comprehended what I have said, and have any confidence in my veracity and mental soundness, I need not say anything more. I could go into the highways and by-ways of the histological and embryological transformations that take place in the mouth and give the apprehensions that have been presented to my own consciousness in endeavoring to comprehend the condition of the situation, but I doubt whether there has been a sufficient amount of interest taken in it, or a sufficiently earnest determination to conquer the difficulty, to do any more than the president said, to be afraid of it. That is because we did not get a sufficient grasp of it to be satisfied that we had the truth.

*Dr. Ottolengui.*—It is not at all necessary for me to substantiate anything that Dr. Atkinson says, because we all know him and believe him, but this case that he has related I saw, through his kindness, and I can say that it was certainly the most remarkable thing that I ever saw, in view of its first condition and the permanent results. It was about that case I was thinking when I asked what the treatment had been in these cases, because I was desirous of bringing out the treatment used by others. It will pay any man to go down to Dr. Atkinson's and see the casts of that case. I feel very grateful to him indeed for allowing me to see the case day after day. Not the least remarkable result was the improvement in the general health of the patient, from a state of nervous prostration and anæmia to one of general good appetite and health,—normal reaction.

*Dr. Allan.*—Mr. President, I want to ask a question. Cannot that adenoid hypertrophy be a result, as well as a cause, of mouth-breathing? Is it not just as liable to be one as the other?

*Dr. Delavan.*—Mr. President, the question of adenoid hypertrophy of the pharynx has now been studied for about twenty years. In 1868, Professor Meyer wrote an article in which he first called direct attention to the importance of this question. It was known long ago that there existed collections of adenoid tissue in the vault of the pharynx, but not until Dr. Meyer's time was its importance recognized. The influences of adenoid hypertrophy upon the well-being of the child was explained more or less fully by Dr. Meyer. They have since been undergoing constant investigation, and many new facts have been added. It is now a question not of the study of an occasional case, but of the observation which has been going on for twenty years in the hands of men in this country and in Europe, upon thousands and even tens of thousands of patients. What has been stated here has been learned through accumulated facts and statistics. The literature of this subject is large, and constantly increasing. To answer the question of the last speaker directly, it is universally held, so far as I know, that the adenoid hypertrophy is the cause of mouth-breathing. Nor can I conceive of any good reason why the contrary should be the case. It is a very difficult thing to settle problems such as confront us in these cases of deformity of the hard palate. The only way by which our knowledge of the subject can be advanced is by the careful study of the cases which present themselves, and the comparison of ideas through such open discussion as we have had to-night. I have not sought to claim anything, but have simply laid before you certain facts as concisely as I could, and have presented such views as have already been recorded as clearly as possible, hoping that, through comparison of experience and opinion, something of advantage might finally be learned.

As to the various causes stated, they are entirely hypothetical, and possibly none of them may be true; they are the best, however, that have thus far been advanced.

With regard to the question raised as to the influence of the tongue upon the deformities of the hard palate, it is undoubtedly true that for the proper development of the mouth the tongue should be in its normal situation, and if it drop with the lower jaw, certainly the development of the roof of the mouth will suffer from its absence.

I should have explained, perhaps, more fully the nature of adenoid growths. Adenoid growths, such as I refer to, are confined mostly to the roof of the pharynx or to the uppermost portion of the posterior wall of the pharynx. They do not descend to the middle of the pharynx, nor do they extend to the epiglottis. An adenoid of fairly good size would be about the size of an enlarged tonsil. Tonsils can be removed with the fingers, but we have better means of operating on them. I have performed operations where the adenoid growths were so soft that with one stroke of the instrument they came away, although a large mass was present. On the other hand, such growths are sometimes very firm, and the older the child the firmer they are apt to be. I have found them in a child, as young as six years of age, so exceedingly dense and tough that no human finger could have removed them.

In view of these facts, since the question of methods of operating has been raised,—although I did not intend to touch upon that at all this evening,—I will say that it is not altogether an easy or simple matter to extract these growths in some of the more important cases that come to us. The operation is a painful one, and without an anæsthetic the child is apt to be severely frightened as well as hurt. Indeed, the effect upon the nervous system is far from beneficial. I have operated since 1878 on these growths, and generally prefer to administer an anæsthetic, unless my patient is pretty well developed, unless he is over fourteen years of age, and unless the growth is small, it is much better that an anæsthetic be used.

In the first place, unless the growth be thoroughly removed the trouble will not be satisfactorily relieved; and in the second place, in order to perform the operation thoroughly and easily, it is necessary that the patient should be in a certain position, and that the services of a trained assistant be employed.

With regard to mouth-breathing, it is not every case of adenoid hypertrophy which is associated with it. A very large adenoid growth in the pharnyx may sometimes be found where the patient is not a mouth-breather, and where there is no deformity of the dental arch or the hard palate. This seems good proof of the fact that occlusion of the nose is the cause of mouth-breathing, and that it has something to do with the deformity of the palate. Where the inspiratory function is maintained through the nose we do not find it deformed or ill developed.

The operation of removing adenoid growths I consider an important one, and it is my practice to keep young patients in bed, sometimes for two days, and to place them at once upon a good course of tonic treatment, and guard them carefully until healing has taken place. It is remarkable to observe the change in the condition of the patient upon the removal of a large adenoid growth. A child, who has been operated upon, will receive, the instant the growth is taken away, a large accession of oxygen, the effect of which is sometimes very exhilarating. The improvement is so plainly evident that the child knows it and feels it. I have known a child of four years old to call attention to improved symptoms which it did not seem possible that one of that age could notice.

I want to say that the object of this paper was to call attention to the connection of adenoid growths with deformity of the hard palate. We are far from knowing all that can be learned about it. But you know more about it, probably, than any others, because you are constantly thrown in contact with this class of patients. When a patient comes to me with projecting front teeth and this high arch palate, I invariably expect to find adenoid hypertrophy of the pharynx, and am rarely disappointed. A patient is sure to suffer harm from the presence of adenoid during the period of constructive activity, no matter if it does disappear when they are grown; then the harm is done, the hearing is impaired, the arch is deformed, and various conditions have become more or less irremediable. It is therefore important that these growths should be removed as early as possible during childhood. The earliest case operated upon that I know of was one of Dr. Hooper's, a child of eight months. I have also operated upon a child of eight months, and also upon several children who were not over fourteen months old, and always with beneficial results.

An infant at the breast nursing is in a very uncomfortable position when it has an adenoid growth that prevents it from breathing through the nose. In sucking, a child must breathe through the nose, or he must drop the nipple to breathe through the mouth. Therefore it is never too early to recognize the trouble; and I can assure you, from the experience of hundreds of observers, that it is very necessary to relieve it at the earliest possible time.

The means by which we operate vary. Time is lacking to describe them, and it is not the object of my paper to do so.

The best way, it seems to me, is to first recognize the condition thoroughly; then to find out what operation is necessary, and the very moment that it can be reasonably made, perform it.

This is a trouble that must often come within the range of your observation, and I think our discussion to-night will tend to direct a little more interest to it, and perhaps throw some light upon it.

*Dr. Niles.*—In regard to the tendency to absorption, I would like to ask Professor Delavan just how far we should be influenced in recommending operations for the removal of adenoid growths; whether in his opinion there are cases which should not be operated upon, trusting to absorption later in life? Dr. Hooper, I think, operated in a case he showed me where the patient was twenty-four years of age, and I think he exhibited another case of a six-months-old infant on which he operated.

*Dr. Delavan.*—The question is a very important one. The age varies greatly. I have a patient under treatment now aged fifty-four years, and I have operated on a number who were over forty. I have never operated on a patient as young as six months. These cases, Meyer says, may be treated with success locally in some instances. In some, constitutional treatment—the administration of cod-liver oil and sending the patient to a better climate, and making applications, such as iodine, to the vault of the pharynx—may do away with the growth. The cases, however, are exceptional.

There are three varieties of the growth,—*first*, those which need not be operated upon ; *second*, a class which may yield to treatment and may not, and which, after treatment for a reasonable length of time, should not be looked upon as curable in that way, but in which further treatment would be time wasted,—operating should be adopted ; and, *third*, a class upon which any local treatment will not have the slightest effect. In these cases it is a waste of time and money to the patients to temporize with them ; they will have to be operated upon sooner or later, and the sooner, under favorable conditions, the better.

*Dr. Dwinelle.*—I feel that we are under great obligations to the doctor for the very interesting entertainment he has given us this evening, and I will move that the thanks of the Society be extended to Dr. Delavan for the very able, interesting, and entertaining essay of the evening.

Dr. Dwinelle's motion was carried.

Adjourned.

S. E. DAVENPORT, D.D.S., M.D.S.,
*Editor New York Odontological Society.*

